# Beyond buzzwords: NLP reveals common threads in sustainable and circular construction discourse

**DOI:** 10.7717/peerj-cs.3085

**Published:** 2025-07-31

**Authors:** Shakarim Aubakirov, Alexandr Pak, Iskander Akhmetov, Aidana Tleuken, Huseyin Atakan Varol, Assel Akzhalova, Ferhat Karaca

**Affiliations:** 1 Kazakh-British Technical University, Almaty, Kazakhstan; 2 Institute of Information and Computational Technologies, Almaty, Kazakhstan; 3 School of Engineering and Digital Sciences, Nazarbayev University, Astana, Kazakhstan; 4 Department Industrial Engineering and Business Information Systems, University of Twente, Enschede, Netherlands; 5 Institute of Smart Systems and Artificial Intelligence, Nazarbayev University, Astana, Kazakhstan

**Keywords:** NLP, Sustainability, Circular economy, Construction, Textrank, TF-IDF, Semantic annotation, Concept analysis

## Abstract

Circular economy and sustainability have both seen rapid growth in academic literature, often leading to ambiguity and the overuse of these terms. This obscures their true objectives and makes it challenging to discern their distinct intentions. Manually analyzing the vast body of recent publications to understand how these concepts connect to environmentally beneficial practices is laborious and time-consuming. This study aims to compare and analyze existing literature on sustainable and circular construction using natural language processing (NLP) techniques to elucidate the similarities and overlaps between these concepts within the construction industry. To achieve this, we employed three NLP methods: (1) TextRank, a graph-based ranking algorithm that extracts key structural relationships between terms in a document; (2) term frequency–inverse document frequency, a statistical measure that identifies the most significant terms based on their frequency and uniqueness within the *corpus*; and (3) semantic annotation (Wikifier), a method that links text tokens to structured knowledge bases such as Wikipedia for better contextual understanding. These methods are used to analyze a dataset of 480 academic articles focusing on sustainability and circular economy in the construction sector. Our analysis revealed that circular construction is more specific and practical, emphasizing resource efficiency, waste management, and industry-specific processes, targeting the operational aspects of recycling and resource recovery. In contrast, sustainable construction encompasses a broader and more holistic scope, including urban planning, community development, and long-term environmental impacts. This study demonstrates how NLP methods can systematically disentangle closely related frameworks in construction literature, providing a replicable methodological framework for future data-driven investigations. By clarifying the distinctions and overlaps between the terms “circular construction” and “sustainable construction”, our research offers enhanced understanding for policymakers, industry practitioners, and academics aiming to integrate sustainable and circular principles effectively within the construction sector.

## Introduction

The construction industry substantially influences environmental changes, specifically global warming (Charef, Lu & Hall, 2022). Therefore, sustainable development and circular economy (CE) in architecture, engineering, and construction sectors are emerging global topics for researchers (Charef, Lu & Hall, 2022; Norouzi et al., 2021; Geissdoerfer et al., 2017). CE emphasizes designing out waste and pollution, keeping products and materials in use, and regenerating natural systems. This contrasts with the traditional linear ‘take-make-dispose’ model, which leads to resource depletion and environmental degradation. Sustainability encompasses a framework that aims to meet present needs without compromising future generations’ ability to meet their own needs (Costa & Rodrigues, 2023). It integrates environmental, social, and economic dimensions to promote long-term ecological balance, social equity, and economic prosperity.

**Figure 1:**
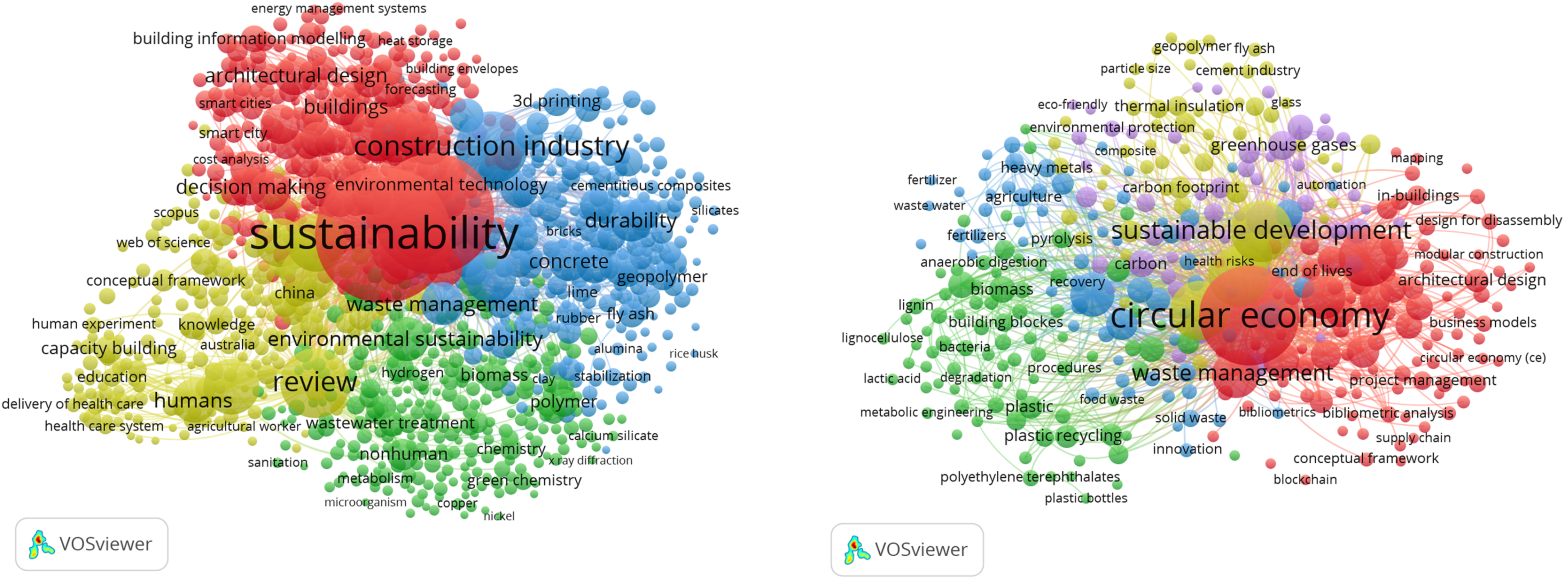
Keywords bibliometric clouds of the “circular economy & construction” and “sustainability & construction”—related academic literature, generated using VOSviewer on the academic dataset used in this study.

Academic interest is growing in these concepts, reflecting their critical roles in fostering a more resilient future. Figure 1 presents bibliometric keyword clouds for academic literature related to “circular economy & construction” and “sustainability & construction.” These provide a visual comparison of distinct yet overlapping themes within the academic literature. Researchers are increasingly exploring innovative strategies to integrate circular and sustainable principles across various sectors, including construction, demonstrating the importance of these concepts and the need for deeper understanding of their applications.

Yet, the concepts of “CE” and “sustainability” might be ambiguous in terms of their similarities and differences (Kirchherr, Reike & Hekkert, 2017). Both refer to ecologically friendly methodologies, however, how to identify the border between these terms, and how can “Circular Construction” and “Sustainable Construction” be specifically defined? In the construction sector, “sustainability” and “CE” might be used interchangeably as buzzwords. The fundamental similarities combining seemingly disconnected “sustainable” and “circular” practices are frequently overlooked, possibly dictated by modern “green fashion” requirements. To improve brand image and demonstrate commitment to sustainability, companies use “sustainability” and “CE” without understanding each concept’s profound significance. Scholars also use sustainability and CE as buzzwords, causing academic confusion rather than real contributions to science (Kirchherr, 2022). The terms “CE” and “sustainability” can be employed as decorative language rather than actual objectives. This approach risks misunderstanding these terms’ significance and implementation of practices crucial for significant environmental benefits.

Applying natural language processing (NLP) methodologies to analyze scientific literature has been widely explored across multiple domains, demonstrating their ability to extract key insights from vast textual corpora. For instance, Panichella (2015) compared traditional text analysis with NLP-driven approaches in software engineering, revealing that NLP provides more structured and context-aware classification of textual data. Similarly, Beasley & Manda (2018) leveraged NLP techniques to automate Gene Ontology term annotation in biomedical research, enhancing accuracy and reducing manual effort for large-scale literature reviews. These studies highlight NLP’s effectiveness in identifying underlying thematic structures and conceptual relations within scientific texts, a methodology adaptable to analyzing intersections and divergences between circular and sustainable construction.

Beyond NLP, bibliometric analysis has also played a crucial role in mapping research trends and conceptual relationships across disciplines. Huang et al. (2024) employed bibliometric methods to conduct a 20-year analysis of anterior segment optical coherence tomography in glaucoma research, mapping scholarly collaborations and keyword distributions to assess field evolution. Mihai, Ulman & Pop (2024) performed bibliometric analysis of scientific publications related to macro- and microplastic pollution, correlating research trends with circular economy principles. These studies demonstrate how bibliometric tools, when combined with NLP, can systematically evaluate large bodies of literature and uncover hidden conceptual connections, offering deeper insights into academic discourse.

Applying this combined methodology to sustainable and circular construction offers a novel research approach. Unlike traditional literature reviews relying on qualitative assessments, NLP-powered bibliometric analysis enables systematic extraction of recurring themes, terminology overlaps, and semantic variations in how sustainability and circular economy concepts are discussed in academic discourse. This approach minimizes subjective bias, enhances analytical accuracy, and provides a data-driven perspective on how these concepts are framed within the construction sector. Prior research in smart cities and urban development has showcased the efficacy of such techniques (da Silva, 2023). By leveraging NLP and bibliometric methods, this study aims to move beyond conventional “green buzzwords” and provide evidence-based understanding of the linguistic and conceptual interplay between sustainability and circular economy in construction research.

Manually analyzing the extensive literature on circular and sustainable construction presents significant challenges. The sheer volume of publications makes it laborious and time-consuming to identify relevant connections to environmentally beneficial practices. This manual process is prone to inconsistencies and may overlook critical insights due to human error and cognitive biases. Consequently, there is a pressing need for a more systematic and efficient approach to literature analysis. By using NLP techniques, researchers can automate the extraction and comparison of key concepts, ensuring comprehensive and unbiased understanding of how circular and sustainable practices intersect within the construction industry. This systematic approach saves time while enhancing analysis accuracy and reliability, providing valuable insights for policymakers, industry practitioners, and academics. The integration of NLP techniques to analyze textual data on circular construction and SC represents a novel approach. To date, no studies have specifically explored similarities and differences between CE and sustainability concepts in the construction industry, particularly through NLP application.

In this context, the following research question arises: What are the differences and similarities between “CE” and “sustainability” concepts in the construction sector? This research article aims to compare and analyze existing literature on sustainable and circular construction utilizing NLP techniques. The primary objective is to identify and elucidate similarities and overlaps between these two significant concepts within the construction industry. Through comprehensive NLP-driven textual analysis, the study aims to find common and diverse themes, terminologies, and approaches that unite and separate both sustainable and circular construction literature, providing deeper understanding of how these concepts intersect and complement each other.

Unlike traditional literature reviews that qualitatively assess sustainability and circular economy in construction, NLP techniques can systematically analyze large *corpus* of academic texts, uncovering recurring themes and terminologies and highlighting linguistic and conceptual overlaps between sustainability and CE in construction. This NLP-driven approach provides evidence-based insights on moving beyond mere “green buzzwords” toward environmental resilience in the construction domain.

## Background

### Circular and sustainable construction

In academia, precise use of scientific terms is essential for clear communication among scientists from various fields (Rheude et al., 2021). Sustainable development and CE in construction frequently co-occur in academic literature (Norouzi et al., 2021; Antwi-Afari, Ng & Hossain, 2021; Mirzyńska et al., 2021), showing their conceptual interrelation and closeness. The overlap of these concepts is confirmed in Sauvé, Bernard & Sloan (2016). Generally, literature shows that CE aligns with sustainable development goals, while, apart from making product lifecycle paths closed-loop, CE’s scope is limited and needs to encompass broader dimensions, such as environmental, economic, and social pillars (Muñoz, Hosseini & Crawford, 2023; Mahanty et al., 2021). Huovila & Westerholm (2022) emphasize that circularity is a strategic approach to achieving sustainable development. Geissdoerfer et al. (2017) considered CE as an essential prerequisite for sustainable development.

However, CE’s social benefits were somewhat limited, predominantly focusing on job creation. Some works have elucidated definitions of environmentally friendly practices in the construction industry (Charef, Lu & Hall, 2022; Rheude et al., 2021; Hasheminasab et al., 2022; Ossio, Salinas & Hernández, 2023). Other scholars suggest that specific SC methods may not align with the CE framework. For instance, Charef, Lu & Hall (2022) stated that “design for” approaches might not be considered truly circular, as reuse potential of some materials is not infinite, thereby not conforming to the closed-loop principle. However, some approaches, such as “design for disassembly,” can still be useful for implementing CE principles in construction. Such strategies help extract and reuse materials efficiently at the end of a building’s life cycle, reducing waste and promoting more circular resource flow. Furthermore, SC methods often include durability and adaptability principles, which support CE’s aim of reducing resource consumption and waste generation. While Charef, Lu & Hall’s (2022) critique is valid for a perspective focusing on “material lifecycle,” it’s crucial to recognize the broader benefits and potential of SC practices at the “project lifecycle” level in advancing CE principles.

In contrast, Kirchherr, Reike & Hekkert (2017) discovered that definitions demonstrate limited direct connections between CE and sustainable development. In another study, it is argued that sustainable development and CE diverge in their fundamental objectives: sustainable development focuses primarily on societal goals, whereas CE centers on altering traditional production and consumption models (Sauvé, Bernard & Sloan, 2016). CE, through its focus on production and consumption activities, prevents negative environmental effects on other sectors, thus helping achieve sustainable development objectives. Moreover, Costa & Rodrigues (2023) claimed that CE largely depends on participant willingness and market-driven solutions, whereas sustainability demands stronger involvement from governments and policymakers.

Luo, Zhuo & Xu (2024) tie optimal NGO staffing to stronger circular-economy governance; Qiao et al. (2024) show sustainability drives knowledge evolution in inclusive tourism; Shi, Hayat & Cai (2024) unveil a unified open-vocabulary network for dense visual prediction; Bibi et al. (2023) optimise low-cost seismic isolation for masonry; Hu et al. (2024) map China’s rare-earth innovation network and its governance-market nexus. Collectively, they reinforce our interdisciplinary, resource-efficient approach to sustainable construction.

### Natural language processing

NLP is a field of artificial intelligence (AI) that focuses on interaction between computers and humans through natural language. NLP has roots in linguistics, computer science and AI, and involves various tasks such as text analysis, machine translation, sentiment analysis, and information retrieval (Sawicki, Ganzha & Paprzycki, 2023). A primary application of NLP is content analysis, where it can uncover patterns, themes, and insights from unstructured text data. Techniques such as tokenisation and stop-word removal are crucial in this stage (Torbarina et al., 2023). After preprocessing, more advanced methods, such as part-of-speech tagging, named entity recognition, and syntactic parsing, are employed to extract meaningful features from text (Chung et al., 2023). NLP techniques can analyse academic articles, industry reports, and news articles to identify common themes and terminologies (Kesgin & Amasyali, 2024). Recent advances in NLP, such as transformer models like BERT and GPT, have significantly enhanced the accuracy and efficiency of text analysis tasks (Torbarina et al., 2023). These models leverage deep learning to understand context and semantics thoroughly, allowing for more nuanced and sophisticated analyses.

Bibliometric analyses have also been employed to study CE integration in the built environment, highlighting key thematic developments and challenges (Mhlanga, Haupt & Loggia, 2024). Another bibliometric research has provided a conceptual framework for understanding the role of CE in the built environment, mapping key research trends and influential publications (Silva Martins et al., 2024). Similarly, scientometric reviews have analyzed sustainability and sustainable development trends, providing insight into the evolution of SC research across multiple disciplines (Olawumi & Chan, 2018). Despite this focus, significant gaps remain in the practical understanding of circular economy and sustainability differences and commonalities in the construction industry.

## Data

This section provides an overview of the data collection and preparation process, outlining criteria and methods used to select relevant literature sources, focusing on key concepts within the CE and SC fields. Additionally, the section details the initial volume, composition, and textual statistics of the collected data. Finally, data preprocessing steps, including cleaning and formatting procedures, are described to ensure dataset integrity and suitability for subsequent NLP analysis.

### Criteria for selecting literature sources for analysis

**Figure 2:**
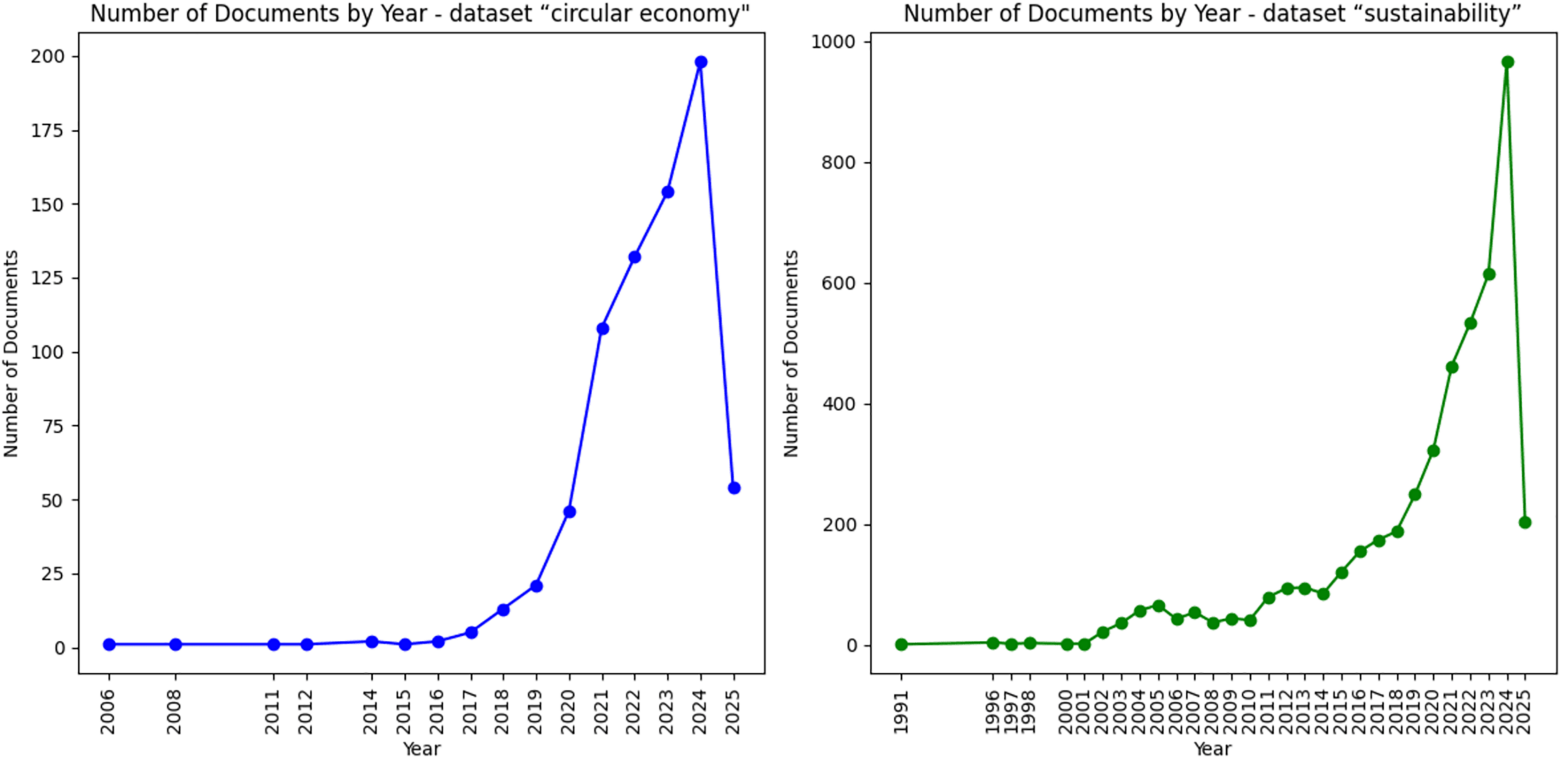
Number of documents by year in “circular economy” and “sustainability” datasets.

Data was gathered through a systematic literature review, employing two distinct searches within the Scopus database. The first search utilised keywords “circular AND economy AND construction OR buildings”, whereas the second focused on “sustainability AND construction OR buildings”. Both searches were confined to review articles published in the last three years (2021–2024). Figure 2 illustrates substantial growth in publications over recent years for both keyword searches. This trend demonstrates increasing attention these topics have received in academic research, particularly after 2020. The rapid surge in review publications highlights emerging interest in synthesising existing knowledge to address practical challenges and knowledge gaps. By focusing on the 2021–2024 period, our study captures this peak of scholarly activity and ensures relevance to the most current developments in circular economy and sustainability within the construction sector. We focused on the 2021–2024 timeframe to capture recent advancements and trends in circular economy and sustainability, ensuring our analysis reflects contemporary practices and terminologies. This approach also allowed effective dataset management while maintaining analytical depth using selected NLP methods. This strategy yielded 400 results for the first query and 1,626 for the second. To ensure relevance to the research topic, each article was carefully evaluated through thorough reading of its abstract. This process led to selecting 107 documents from the first query and 373 documents from the second query, deemed suitable for further NLP data analysis.

### Data collection

After collecting data in PDF and HTML formats, conversion to a usable form was required. PDFReader (Maupin, 2018), Apache Tika (The Apache Software Foundation, 2024), and PDFMiner (Shinyama & Guglielmetti, 2023) were selected to handle PDF articles due to their robust text extraction capabilities and handling of complex document structures. Table 1 presents a summarised description of each tool. Multiple tools were utilised because certain PDF files had specific characteristics preventing a single method from extracting information effectively. Beautiful Soup (Richardson, 2023) was chosen for HTML files due to its effective HTML parsing and content extraction. We parsed 381 PDF and HTML files and successfully extracted the necessary texts for analysis. Each sample represents a single research article, providing a foundation for subsequent analysis. This dataset, titled “Sustainable and Circular Construction Terms”, was published Karaca et al. (2024).

**Table 1:** Tools and descriptions.

Tool name	Description
PdfReader	A lightweight Python library used to read PDF files and extract text. It is simple to use and works well with straightforward PDFs.
Apache Tika	A content analysis toolkit that extracts text and metadata from various document formats, including PDFs. It is particularly useful for handling a wide range of document types and ensuring accurate text.
PDFMiner	A powerful tool for extracting text, images, and other data from PDF documents. It is highly effective in dealing with complex document structures, such as multi-column layouts and embedded fonts.
Beautiful Soup	A Python library that effectively parses HTML and extracts relevant content. It creates parse trees that help extract the necessary information from web pages efficiently.

Upon constructing our dataset, we organised it into a structured format. The dataset includes the following components: author information, paper title, abstract text, article text (in two representations: (1) a contiguous text block containing the entire article and (2) a segmented list of individual sentences), and quantitative metrics (length and complexity of each article, measuring the number of symbols, words, and sentences).

### Data cleaning

Data cleaning procedures were essential to minimise noise and enhance the accuracy of subsequent text analyses, ensuring the dataset was structured and clean. This section describes the steps to refine the collected dataset. To focus analysis on core article content, we removed abstracts from the **article_text_string’** since they were already stored separately in **abstract’**, making their presence redundant and potentially skewing text analysis metrics such as word and sentence counts. Similarly, all embedded URLs were removed as they are non-informative for textual analysis, ensuring subsequent studies, like frequency distributions or topic modelling, are based solely on substantive content.

Another cleaning step involved extracting and isolating references from each document. References indicate research depth and breadth but are peripheral to primary text analysis. By storing references in a dedicated ‘references’ column, we maintained access to this data without cluttering the primary text analysis fields.

### Data preprocessing

Following the initial data cleaning phase, we conducted data preprocessing to refine our dataset to transform the raw text data into a structured and analytically processable format.

We removed all numerical digits from article texts during preprocessing to focus analysis on textual content and avoid the potential bias that numbers might introduce to text analysis metrics. We also removed mathematical symbols, which could similarly skew text analytics results by being mistaken for textual content.

These elements—numerical values, units, and equations—often reflect dataset-specific noise rather than consistent semantic meaning across documents. Since our study aims to extract thematic and conceptual patterns rather than quantitative analysis, retaining such elements would distort frequency-based metrics (TF-IDF) and introduce irrelevant nodes into graph-based models (TextRank). Their exclusion ensured that term extraction focused solely on meaningful linguistic constructs.

Another significant preprocessing step was removing stop-words from each article text. Stop-words—commonly used words with minimal individual meaning—contribute little to understanding text content and can disproportionately affect text statistics (Manning, Raghavan & Schütze, 2008). Their elimination enhances focus on relevant vocabulary within text mining.

We used the default English stop-word list from the Natural Language Toolkit (NLTK) library, which offers a balanced set of commonly non-informative terms suitable for academic discourse. We intentionally did not apply stemming or lemmatisation to preserve original lexical forms of technical and domain-specific terms (*e.g*., “recycling,” “recycled materials,” “resource recovery”), which might otherwise be distorted. This decision ensured higher fidelity in concept recognition and maintained semantic granularity important for later clustering and semantic annotation.

After these preprocessing steps, the statistics (the number of words, symbols, and sentences) for each article were calculated (see Table 2). Table 2 represents the descriptive statistics of quantitative metrics for number of symbols, words, and sentences in the collected data *corpus*.

**Table 2:** Descriptive statistics of quantitative metrics.

	num_symbols	num_words	num_sentences
mean	31,981.35	4,017.48	388.93
std	27,098.02	3,493.10	383.06
min	579.00	55.00	1.00
25%	8,188.50	999.00	86.50
50%	27,692.00	3,459.00	304.00
75%	50,061.00	6,227.50	554.50
max	157,952.00	21,209.00	2,192.00

**Figure 3:**
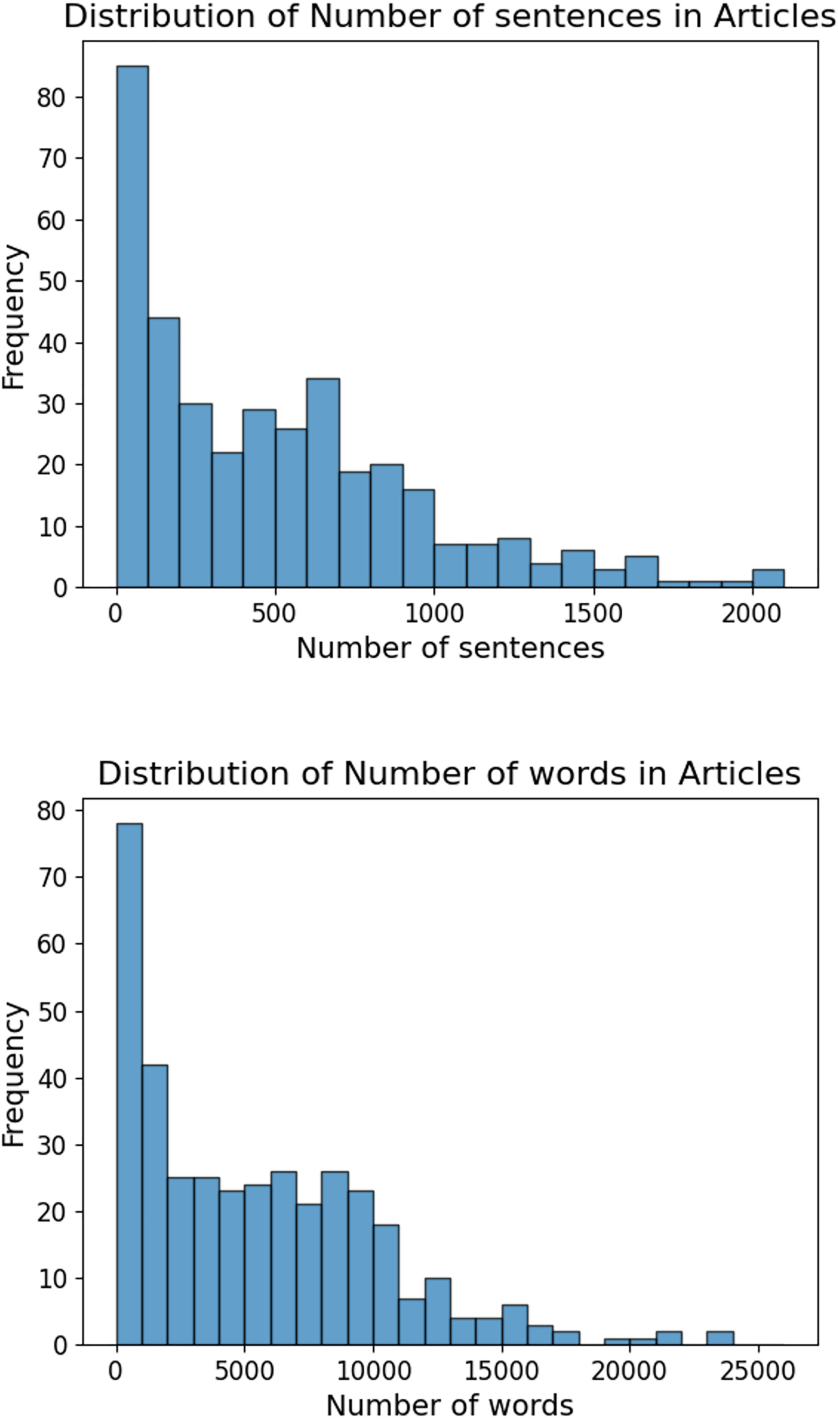
Distribution of sentences and words: the graph shows the number of sentences and number of words after preprocessing.

The histograms in Fig. 3 demonstrate the distribution of sentences and words in the articles after preprocessing. Understanding these distributions helps identify the general structure and length of the articles (especially by looking at the number of words since we are going to find the most valuable terms from the articles), which can influence the effectiveness of the subsequent NLP analysis. The histogram for the number of sentences shows a right-skewed distribution, with a high frequency of articles having fewer sentences and a tail extending towards articles with more sentences. This indicates that while most articles are concise, a few are significantly longer. Similarly, the histogram for the number of words is also right-skewed, with most articles having a lower word count and fewer articles being lengthy. This skewness suggests a prevalence of shorter articles in the dataset, with a small proportion being more detailed.

**Figure 4:**
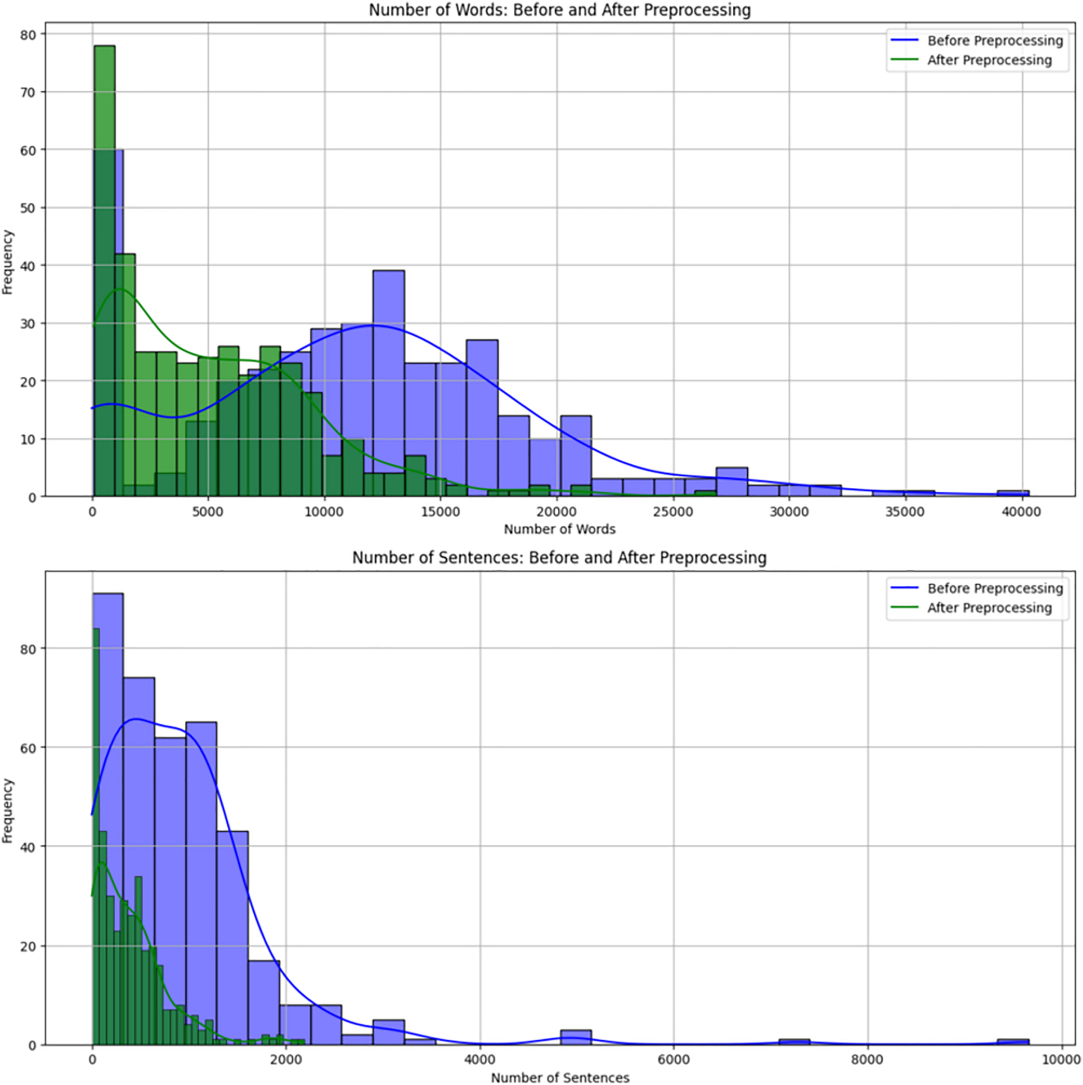
Comparison of word and sentence count distributions before and after preprocessing. The histograms show how preprocessing refines the dataset by reducing irrelevant content, leading to more standardised and structured text data.

To further illustrate the impact of preprocessing, Fig. 4 presents a comparative distribution of word and sentence counts before and after preprocessing. The histogram highlights how the text cleaning process, including stop-word removal, numerical filtering, and symbol elimination, affected the dataset. The shift in distributions demonstrates that preprocessing significantly reduced text length while maintaining the overall structure. This comparison validates the necessity of preprocessing, as it ensures that the data used for further NLP analysis is more refined and free from non-informative elements.

## Methodology

**Figure 5:**
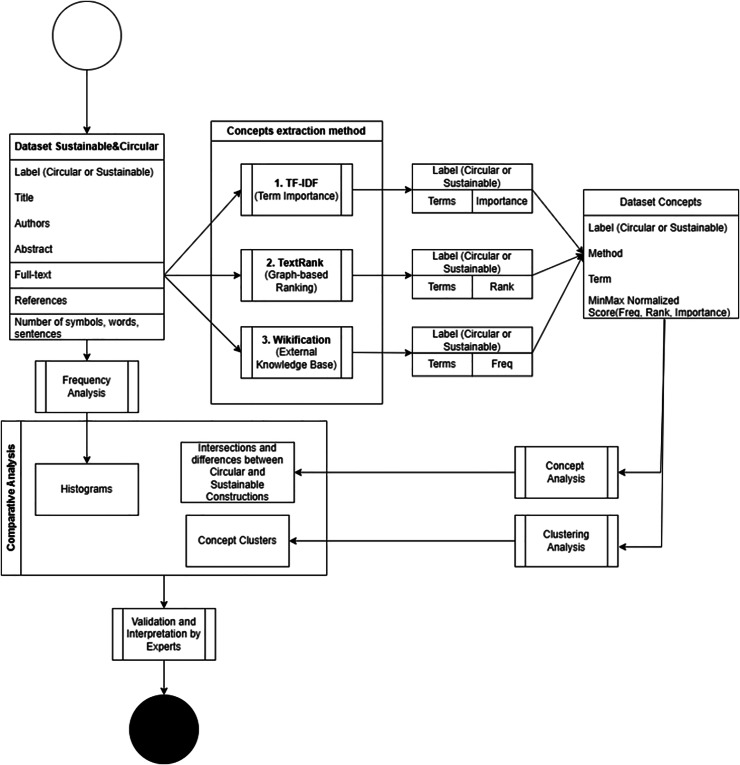
Flowchart of data analysis.

The methodology adopted for this study encompasses an approach to analyse textual data related to sustainability (SC) and circular construction (CC) within the construction sector. The methods combine state-of-the-art NLP techniques with robust analytical tools to extract, cluster, and interpret significant terms from the data. By employing a mix of statistical, graph-based, and conceptual analysis frameworks, we aim to derive meaningful insights and thematic representations of the textual data. The following sections detail the specific techniques and tools utilised, as well as the evaluation methods applied to assess the results. Figure 5 provides an overview of the research methodology. Bibliometric and scientometric approaches have been widely used to map research trends in CE and sustainability (Asare, Obi & Thurairajah, 2024; Ece Kaya & Erbaş, 2024).

### Description of the used NLP techniques and tools

To analyze textual data related to SC and CC in the construction sector, we employed three complementary NLP techniques: TF-IDF, a statistical method that quantifies term importance based on document frequency (Rajaraman & Ullman, 2011); TextRank, a graph-based ranking algorithm inspired by PageRank that extracts keywords through term co-occurrence relationships (Page et al., 1999); and semantic annotation, a knowledge-based approach linking text tokens to external knowledge sources like Wikipedia (Brank, Leban & Grobelnik, 2018; Klopper & Lubbe, 2012). Studies such as Obi et al. (2022) have demonstrated that automated text-mining techniques effectively identify key sustainability and CE trends in construction research. These methods complement each other: TF-IDF identifies statistically discriminative terms, TextRank captures term relationships through graph structures, and semantic annotation introduces external contextual knowledge for term disambiguation and enrichment.

#### TF-IDF

TF-IDF evaluates word importance in a document relative to a *corpus* (Rajaraman & Ullman, 2011), combining term frequency (TF) and inverse document frequency (IDF) (Luhn, 1957). The score increases with word frequency in a document but is offset by *corpus* frequency, highlighting significant terms within specific documents. Using TfidfVectorizer from sklearn (Pedregosa et al., 2018), we transformed text into a TF-IDF matrix where rows represent documents and columns represent terms. TF-IDF weights were calculated for each term, and top terms were selected based on threshold values for balanced comparison.

#### TextRank

TextRank is an unsupervised graph-based algorithm inspired by PageRank for keyword extraction (Page et al., 1999). It constructs a graph where the vertices represent words and edges represent co-occurrences within a sliding window. Word importance is calculated based on connections, identifying the most significant terms. We constructed a word graph with nodes representing words and edges representing co-occurrences (Mihalcea & Tarau, 2004), applied PageRank to calculate word importance (Page et al., 1999), and selected top-ranked terms based on threshold values. After extracting key terms, we performed clustering using a pre-trained Bidirectional Encoder Representations from Transformers (BERT) model (Devlin et al., 2019) for vectorisation, applied hierarchical clustering with dendrogram visualisation (Kaufman & Rousseeuw, 2009), determined optimal cluster numbers using the elbow method (Thorndike, 1953), and finalised grouping with K-means clustering (MacQueen, 1967). Each cluster was assigned descriptive thematic labels.

#### Semantic annotation

Semantic annotation extracts and organises key ideas from complex texts (Brank, Leban & Grobelnik, 2018). We employed a wikification-based approach using Wikipedia articles as concept representations. The process involved identifying relevant terms corresponding to Wikipedia concepts, constructing a mention-concept graph with nodes representing terms and candidate concepts, and applying global disambiguation using PageRank-based ranking (Mihalcea & Csomai, 2007). We filtered annotations with low semantic relevance and constructed a concept matrix organising terms into categories such as Sustainability, Circular Economy, Built Environment, Economics, and Waste Management. This method enhances interpretability and enables systematic differentiation between sustainability and circular economy themes (Fotova Čiković, Martinčević & Lozić, 2022).

### Evaluation methods

Evaluation used three extraction methods: TF-IDF, TextRank, and Wikification, providing comprehensive concept coverage across scientific articles about sustainable and circular construction. Concept clustering grouped similar terms using vector-based similarity measures, identifying dominant themes and facilitating interpretation. Semantic analysis examined contextual meaning through term co-occurrences and external knowledge base linkage *via* Wikification. Expert evaluation by sustainability and construction specialists validated extracted concepts and associations, ensuring accuracy and relevance of identified themes. This framework provides a systematic analysis of evolving terminologies with reproducibility and structured comparison capabilities.

**Figure 6:**
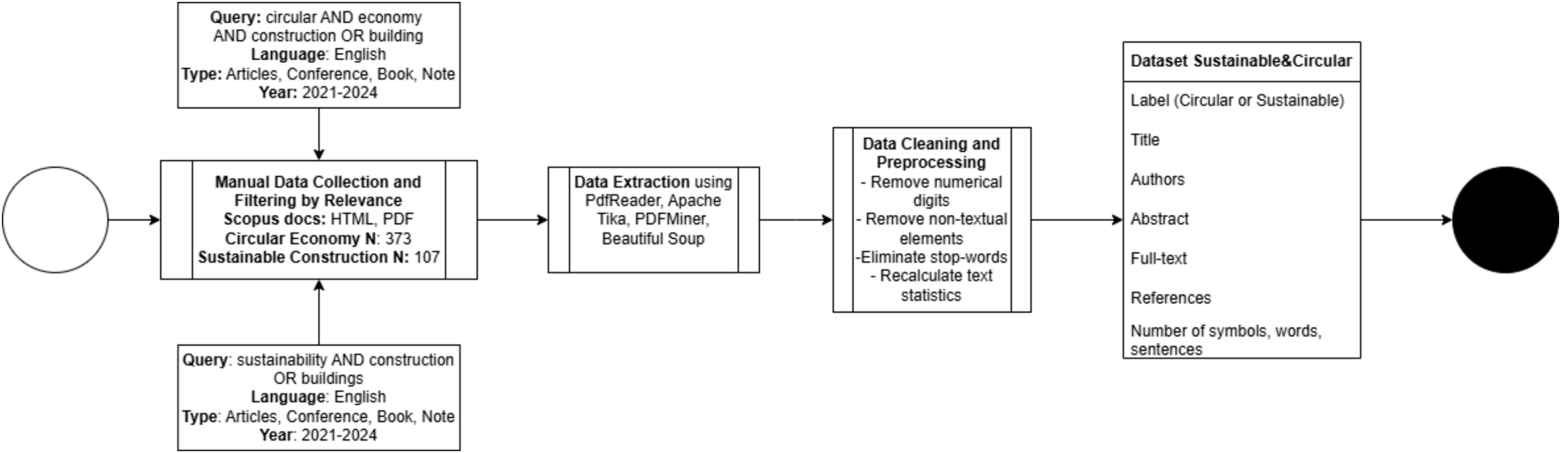
Flowchart of data collection and construction.

The data analysis flowchart in Fig. 5 outlines the analysis procedure, and the data collection and construction flowchart in Fig. 6.

To ensure comparability across extraction methods, we maintained a similar number of clusters (typically 5–7 per method) for TF-IDF, TextRank, and Wikifier outputs. This alignment facilitated thematic comparison across techniques and avoided disproportionate clustering that could hinder cross-method analysis. The number of clusters was guided by the elbow method and refined through interpretability. Cluster labels were manually assigned based on semantic consistency within each group of terms. Furthermore, we assessed the overlap and divergence between methods using Venn diagrams and word cloud visualisations, identifying shared and method-specific terms. This comparative evaluation allowed us to highlight consistent domain concepts and better understand the unique strengths of each extraction technique.

## Results

This section presents the analysis of clusters extracted from two data *corpus* analyses on circular construction (CC) and sustainable construction (SC). First, we will discuss the TF-IDF approach, followed by the TextRank approach, and, finally, the semantic annotation approach. It is followed by the comparison of two concepts using the obtained results.

In the context of this study, we refer to “Circular Construction” refers to the application of circular economy principles to the construction sector, focusing on designing out waste, keeping products and materials in use for as long as possible, and regenerating natural systems. “Sustainable Construction” encompasses the design, building, operation, maintenance, and end-of-life disposal of buildings and infrastructure in a manner that minimally impacts the environment, supports social well-being, and promotes economic viability. This approach considers the entire life cycle of a project, striving to balance environmental protection, resource conservation, and societal needs.

### TF-IDF results and analysis

The results of the term clustering and their comparative analysis of terms related to CC and SC using the TF-IDF method are provided in Table 3. It is seen how CC and SC are similar and differ in terms of their content. To start with similarities, both CC and SC have such themes as “environmental impact assessment”. This indicates a shared concern for understanding and mitigating the negative effects of construction activities on the environment. Environmental impact assessment is crucial for both concepts as it provides a systematic process for evaluating the potential environmental consequences of proposed projects or policies, helping to ensure that such initiatives are sustainable and environmentally responsible.

**Table 3:** Key concepts and themes in CC and SC clusters (TF-IDF analysis).

Field	Key concepts	Themes
Circular construction (CC)	Building deconstruction, resource recovery, zero waste, regenerative design, urban mining, passive house, reverse logistics	Resource management and ecological design
	Construction waste, electronic waste, recycling industry, recycled materials, waste management hierarchy, waste incineration	Waste utilisation and recycling
	Environmental assessment, sustainable building, green building, industrial ecology, structural design, life cycle assessment, reduce reuse recycle	Environmental assessment
Sustainable construction (SC)	Sustainable building, compact city, sustainable food, impact assessment, cost-benefit analysis, conference papers	Sustainable building and impact assessment
	Green buildings, edible landscaping, urban agriculture, timber frame, noise pollution, green building council, public sector	Green building and urban agriculture
	Sustainable cities, urban ecology, urban ecosystems, green building materials, green construction, life cycle assessments, sustainability assessment	Sustainable cities and urban ecology
	Energy-efficient building, green building, timber construction, wood products, renewable energy sources, renewable energy, energy conservation	Energy efficiency and construction materials

Despite these similarities, there are notable differences between the terms. CC dataset is more inclined towards “waste and resource utilisation”, being more focused on “resource efficiency”. This emphasis on optimising resource use, minimising waste, and promoting recycling and reuse highlights CE’s goal of creating closed-loop systems where materials are continuously cycled back into production processes (Korhonen, Honkasalo & Seppälä, 2018).

In contrast, SC emerges as a more foundational and comprehensive concept, comprising a broader scope that extends beyond the building sector to include cities and energy usage. This reflects the three-pillar nature of sustainability, which balances environmental, social, and economic goals. Sustainability in construction encompasses a more holistic approach (Geissdoerfer et al., 2017; Muñoz, Hosseini & Crawford, 2023; Mahanty et al., 2021), contrasting with CC’s more targeted focus on resource cycles within specific industries or sectors.

The comparative analysis using the TF-IDF method further reveals that while CC focuses on practical applications and processes related to waste management and resource efficiency, SC covers a broader vision that integrates aspects of urban planning, community development, and energy management. For instance, terms related to “cities” and “land use” frequently appear in the SC dataset, emphasising its relevance to urban sustainability and regional planning.

#### Circular construction (CC)

The clusters identified in the CC topic revolve around three main themes: resource management and ecological design, waste utilisation and recycling, and environmental assessment in sustainable construction. The first cluster, *Resource Management and Ecological Design*, encompasses terms related to resource recovery, zero waste, regenerative design, and urban mining, emphasising waste minimisation through efficient resource utilisation and innovative design strategies. The second cluster, *Waste Utilisation and Recycling*, focuses on waste management processes and material recycling, with key terms like construction waste, electronic waste, recycling industry, and waste management hierarchy, underscoring structured approaches to waste processing and transforming waste into reusable materials. The third cluster, *Environmental Assessment and Sustainable Construction*, includes environmental assessment, green building, industrial ecology, and structural design. The inclusion of “life cycle assessment” and “reduce, reuse, recycle” highlights strategies for evaluating and improving construction’s environmental footprint while integrating sustainability into building codes and practices. These clusters illustrate essential components of a circular economy approach in the built environment, emphasising sustainability, resource efficiency, and waste reduction.

#### Sustainable construction (SC)

The study of SC can be categorised into four main clusters: sustainable building and impact assessment, green building and urban agriculture, sustainable cities and urban ecology, and energy-efficient building with timber materials. The first cluster, Sustainable Building and Impact Assessment, encompasses sustainable building, compact city, and impact assessment, emphasising environmentally friendly urban development and evaluation of sustainable practices through tools like cost-benefit analysis. The second cluster, Green Building and Urban Agriculture, focuses on integrating green buildings and urban agriculture into city planning, featuring concepts like edible landscaping, timber frame, and noise pollution. Organisations such as the Green Building Council and the public sector highlight various stakeholders’ roles in advancing sustainability initiatives. The third cluster, Sustainable Cities and Urban Ecology, includes sustainable cities, urban ecosystems, and green construction, illustrating efforts to develop urban areas prioritising ecological health and resilience through life cycle assessments and sustainability assessments. The fourth cluster, Energy-Efficient Building and Timber Materials, centres on natural and renewable building materials, highlighting energy-efficient technologies, timber construction, and wood products as key components in reducing buildings’ environmental footprint. These clusters reflect a comprehensive approach to sustainable construction, integrating ecological considerations, urban resilience, energy efficiency, and natural material use into the built environment.

### TextRank results and analysis

TextRank method results for CC and SC key concepts are summarised in Table 4. Based on the obtained results, it is seen that both concepts have energy-related themes; however, their underlying key concepts differ. That is, CC is more focused on resource recovery, while SC emphasises energy-efficient building design and related materials, *e.g*., timber. It is interesting to note that the CC dataset contains the term “sustainability”, while the SC dataset does not contain the term “circularity”, which can be justified by sustainable development being a more fundamental concept than CE. The presence of the term “sustainability” within the CE dataset suggests that CE frameworks often incorporate sustainability principles, reflecting a nested relationship where CE practices contribute to broader sustainability goals (Korhonen, Honkasalo & Seppälä, 2018).

**Table 4:** Key concepts and themes in CC and SC clusters (TextRank analysis).

Field	Key concepts	Themes
Circular construction (CC)	Construction waste, electronic waste, recycled material, recyclable materials, waste recycling, waste hierarchy, organic waste	Waste management and recycling processes
	Recycling industry, renewable energy production, construction operations, mining sector, energy services, construction industry, energy production	Industrial and economic sectors
	Building, buildings, timber construction, flexural strength, construction projects, civil construction, resource use	Building and resource management
	Environmental assessment, recycling technology, life cycle assessment, architectural design, environmental science, sustainability assessment, environmental protection	Environmental and life cycle analysis
	Energy reuse, resource recovery, renewable resources, green buildings, sustainable building, renewable energy, CE, environmental impact	Sustainable practices and energy efficiency
Sustainable construction (SC)	Sustainable building, impact assessment, green building, industrial ecology, structural design, life cycle assessments, resource depletion, environmental degradation	SC and environmental impact
	Green buildings, urban agriculture, timber frames, noise pollution, green deals, land use, green building council, public sector	Urban planning and agricultural practices
	Sustainable cities, urban ecology, ecosystems, green building materials, construction, urban planning, urban development	Urban sustainability and ecological systems
	Energy-efficient building, green building, timber construction, wood products, renewable energy sources, renewable energy, energy conservation	Energy efficiency and construction materials

Both concepts share a theme of assessment and lifecycle analysis. However, CE is more inclined towards the resources lifecycle, focusing on aspects such as recycling, reusing, and minimising waste throughout the production and consumption cycles. On the other hand, sustainability pays more attention to the building lifecycle, including energy consumption, material durability, and overall environmental impact over time. This distinction demonstrates the different scales and scopes at which each concept operates, with CC being more process-oriented and SC being more holistic in its approach to built environments.

The SC concept operates more at a regional level, using keywords like “land use” and “cities.” This regional focus highlights sustainable development’s importance in adopting policies and practices impacting communities, infrastructure, and ecosystems at local and regional scales (Newman & Jennings, 2008; Orenstein & Shach-Pinsley, 2017; Thacker et al., 2019).

In contrast, CC focuses more on industry level, resources, and building use, reflecting practical implementation of circular practices within manufacturing, construction, and other sectors. The differences between SC and CC also imply varying approaches to policy and innovation. SC might drive policy development toward urban sustainability strategies, influencing zoning laws, building codes, standardisation, and transportation planning. Meanwhile, CC could stimulate innovation in product design and industrial processes, encouraging businesses to adopt circular practices.

#### Circular construction (CC)

The concept of CC can be categorised into five key clusters: waste management and recycling processes, industrial and economic sectors, building and resource management, environmental and life cycle analysis, and sustainable practices with energy efficiency. The first cluster, Waste Management and Recycling Processes, focuses on transforming waste into reusable materials through efficient recycling, with key terms such as construction waste, electronic waste, and recyclable materials. Concepts like waste hierarchy and organic waste emphasise structured approaches aimed at minimising environmental impact. The second cluster, Industrial and Economic Sectors, highlights various industries’ role in promoting circular economy practices, featuring the recycling industry, renewable energy production, construction operations, and energy services, underscoring sustainability integration within major economic sectors. The third cluster, Building and Resource Management, covers sustainable building practices and resource efficiency, with terms such as timber construction, flexural strength, and construction projects, reflecting the industry’s commitment to optimising resource use and sustainable design. The fourth cluster, Environmental and Life Cycle Analysis, centres on assessing environmental impacts and implementing sustainable solutions, incorporating environmental assessment, life cycle assessment, recycling technology, and architectural design. The fifth cluster, Sustainable Practices and Energy Efficiency, emphasises energy reuse, resource recovery, and renewable energy adoption, with key concepts including green buildings, sustainable building, and renewable resources. Terms like circular economy (CE) and environmental impact reflect a holistic approach integrating resource management, sustainability, and energy efficiency into construction practices. These clusters illustrate a comprehensive framework for circular construction, emphasising waste reduction, industrial collaboration, sustainable building, environmental responsibility, and energy-efficient innovations.

#### Sustainable construction (SC)

The field of SC can be categorised into four key clusters: SC and environmental impact, urban planning and agricultural practices, urban sustainability and ecological systems, and energy efficiency with construction materials. The first cluster, SC and Environmental Impact, focuses on sustainable building practices and their effects, incorporating key terms such as sustainable building, impact assessment, green building, and life cycle assessments. The inclusion of resource depletion and environmental degradation highlights the need for strategies that mitigate negative environmental consequences. The second cluster, Urban Planning and Agricultural Practices, explores the integration of agricultural practices into urban design, with key terms including urban agriculture, timber frames, noise pollution, and land use. The mention of the Green Building Council and public sector underscores the role of various stakeholders in advancing sustainable urban development. The third cluster, Urban Sustainability and Ecological Systems, addresses sustainable cities, urban ecology, ecosystems, and green building materials, reflecting efforts to develop resilient and environmentally friendly urban spaces. Terms such as urban planning and urban development emphasise the importance of sustainable approaches in shaping future cities. Lastly, the fourth cluster, Energy Efficiency and Construction Materials, highlights the role of renewable materials and energy-efficient technologies in construction, with key concepts including energy-efficient buildings, timber construction, wood products, and renewable energy sources. The focus on renewable energy and energy conservation further underscores the need for energy efficiency in SC practices. Collectively, these clusters illustrate a comprehensive approach to sustainable construction, emphasising environmental responsibility, urban resilience, stakeholder engagement, and the adoption of energy-efficient and sustainable building materials.

### Semantic annotations results and analysis

Table 5 presents the results of the semantic annotation method for the analysis of CC and SC key concepts. CC and SC share a focus on economic and technological systems, governance frameworks, and environmental protection. Both emphasise business, methodology, financial instruments, technology, and policy to guide construction practices. They also incorporate building materials, planning, and manufacturing to improve efficiency. However, CC prioritises trade, medium of exchange, alternative currency, and blockchain, highlighting its emphasis on economic value and resource circulation. It also focuses on data storage, software development methodology, and energy storage, underlining its reliance on technological innovation. SC, in contrast, integrates urban planning, infrastructure, logistics, and climate classification, demonstrating a broader concern for long-term environmental impact and societal well-being. SC also highlights environmental change, greenhouse gases, pollutants, and soil conditioners, indicating a stronger focus on ecological health. While CC is centred on optimising material and energy exchange, SC takes a more systemic approach, addressing governance, administrative entities, and political systems.

**Table 5:** Key concepts and themes in CC and SC clusters (semantic annotation analysis).

Field	Key concepts	Themes
Circular construction (CC)	Accounting book, additive, algorithm, analysis, business, economic concept, financial system, methodology, legal instrument, technology, trade, uncertainty, wealth	Economic and technological systems
	Academic degree, behaviour, classification system, concept, design, information, legal form, management, method, policy, principle, rule, standard, system	Knowledge and governance frameworks
	Abstract being, artificial entity, chemical entity, material property, medium of exchange, physical quantity, recording medium, transportable goods	Material and energy exchange
	Academic journal, alternative currency, applied science, blockchain, building material, construction technique, data storage, energy storage, environmental protection, software development methodology	Engineering, innovation, and environmental technologies
Sustainable construction (SC)	Absence, academic discipline, asset, building, business, cognition, competence, efficiency, financial instrument, legislation, manufacturing, methodology, monitoring, network, organisation, planning	Strategic development and governance
	Administrative entity, architectural structure, artistic technique, building material, climate classification, digital media, economic activity, energy policy, infrastructure, logistics, material flow, political system, technology	Urban planning and socioeconomic structures
	Air pollutant, biomolecule, carbonate, chemical substance, disposable product, environmental change, food additive, greenhouse gas, pollutant, soil conditioner	Environmental and chemical dynamics
	Algorithm, artificial geographic entity, baryonic matter, computer network, economic resource, mechanical property, quantum particle, real-valued function, state of matter, structural class of chemicals	Computational and physical systems

Table 6 shows the co-occurrence of concepts of Sustainability and CE. In general, this table is a sign of similarity between both concepts, as there is significant overlap in co-occurrence across all terms. The terms themselves also co-occur frequently, which aligns with existing literature (Norouzi et al., 2021; Mirzyńska et al., 2021; Antwi-Afari, Ng & Hossain, 2021). Talking about differences, it is evident that “Sustainability” more frequently co-occurs with all of the concepts compared to “Circular.” Particularly, examining the field of “Built Environment,” we see a significant difference in co-occurrence, with 0.13 for Sustainability and 0.05 for CE. This discrepancy suggests that sustainability principles are more deeply integrated into building and urban planning practices than the principles of the CE. In almost all of the concepts, the difference in co-occurrence frequency is large; however, in the case of “Waste Management,” it is almost equal. This could indicate that sustainable development is a broader topic than CE but also a fundamental concept that is related to all others.

**Table 6:** Concept matrix of semantic annotation: co-occurrence of concepts in CC and SC datasets, normalized by columns values.

	Sustainability	CE	Built environment	Economics	Environment	General technology	Materials	Science and research	Waste management
Sustainability	–	0.09	0.13	0.12	0.13	0.14	0.14	0.12	0.1
CE	0.04	–	0.05	0.07	0.06	0.05	0.05	0.06	0.08
Built environment	0.17	0.15	–	0.15	0.14	0.19	0.15	0.18	0.13
Economics	0.05	0.06	0.05	–	0.04	0.05	0.02	0.07	0.04
Environment	0.15	0.15	0.13	0.13	–	0.16	0.19	0.13	0.15
General technology	0.25	0.18	0.27	0.21	0.25	–	0.24	0.3	0.22
Materials	0.14	0.1	0.12	0.05	0.17	0.14	–	0.07	0.18
Science and research	0.13	0.14	0.16	0.2	0.12	0.18	0.07	–	0.1
Waste management	0.08	0.13	0.08	0.07	0.09	0.09	0.13	0.07	–

This observation is further supported by the TF-IDF analysis (Table 7), which reveals that terms associated with Sustainable Construction appear more prominently in discussions concerning environmental impact, urban planning, and resource management. The high TF-IDF values for these concepts indicate their greater importance in sustainability discourse when compared to circular economy-related terms. This suggests that while CE is an integral part of sustainability, it remains a subset of a larger and more widely applied framework.

**Table 7:** Concept matrix of TF-IDF: co-occurrence of concepts in CC and SC datasets.

	SC and environmental impact	Urban planning and agricultural practices	Sustainable cities and urban ecology	Energy efficiency and construction materials	Resource management and ecological design	Waste utilization and recycling	Environmental assessment
SC and environmental impact	–	0.13	0.14	0.12	0.06	0.13	0.16
Urban planning and agricultural practices	0.17	–	0.14	0.16	0.19	0.10	0.15
Sustainable cities and urban ecology	0.14	0.11	–	0.11	0.15	0.13	0.08
Energy efficiency and construction materials	0.17	0.18	0.17	–	0.17	0.17	0.18
Resource management and ecological design	0.08	0.19	0.20	0.15	–	0.20	0.25
Waste utilization and recycling	0.13	0.08	0.13	0.12	0.15	–	0.18
Environmental assessment	0.23	0.18	0.12	0.18	0.28	0.26	–

A more detailed perspective on the structural differences between CC and SC is provided by the TextRank analysis (Table 8). This method highlights that key nodes in the SC network are centred around energy efficiency, waste recycling, and life cycle management, reflecting its strong environmental focus. In contrast, CC places greater emphasis on digital technologies, resource exchange, and financial instruments, reinforcing its economic and technological orientation. These differences illustrate that while both frameworks aim for sustainability, they approach it from distinct perspectives: SC takes a more systemic and long-term environmental approach, while CC is driven by innovation and optimisation of economic resources.

**Table 8:** Semantic annotation and TextRank Matrix: co-occurrence of concepts in CC and SC datasets, normalized by columns values.

	SC and environmental impact	Urban planning and agricultural practices	Urban sustainability and ecological systems	Energy efficiency and construction materials	Waste management and recycling processes	Industrial and economic sectors	Building and resource management	Environmental and life cycle analysis	Sustainable practices and energy efficiency
SC and environmental impact	–	0.04	0.04	0.05	0.04	0.04	0.03	0.04	0.05
Urban planning and agricultural practices	0.06	–	0.07	0.06	0.03	0.04	0.09	0.05	0.07
Urban sustainability and ecological systems	0.10	0.11	–	0.12	0.11	0.11	0.05	0.12	0.14
Energy efficiency and construction materials	0.24	0.21	0.27	–	0.22	0.21	0.30	0.19	0.14
Waste management and recycling processes	0.09	0.04	0.10	0.09	–	0.08	0.13	0.08	0.11
Industrial and economic sectors	0.04	0.02	0.04	0.04	0.03	–	0.05	0.03	0.04
Building and resource management	0.18	0.31	0.12	0.32	0.32	0.30	–	0.31	0.40
Environmental and life cycle analysis	0.04	0.03	0.04	0.03	0.03	0.03	0.05	–	0.04
Sustainable practices and energy efficiency	0.21	0.20	0.24	0.11	0.21	0.19	0.30	0.18	–

### Comparative analysis

This analysis aims to highlight the strengths and limitations of each approach and their agreement in identifying key terms related to SC and CE. By leveraging multiple extraction techniques—TF-IDF, TextRank, and Wikifier—we can observe how each method interprets textual importance through statistical frequency, graph-based relationships, or conceptual connections with external knowledge bases. Examining their individual outputs and intersections provides valuable insights into the consistency and uniqueness of extracted terms.

To visualise and quantify overlaps and divergences among these methods, we analyse their intersections using a Venn diagram^1^ 1A Venn diagram is a visual representation of sets, showing their relationships and overlaps. In this case, it illustrates the common and unique terms identified by different extraction methods., followed by exploring word clouds^2^ 2A word cloud is a graphical representation of text data where the size of each word reflects its frequency or importance in the analysed text., which offer an intuitive representation of term prominence within each approach.

#### Venn diagram

**Figure 7:**
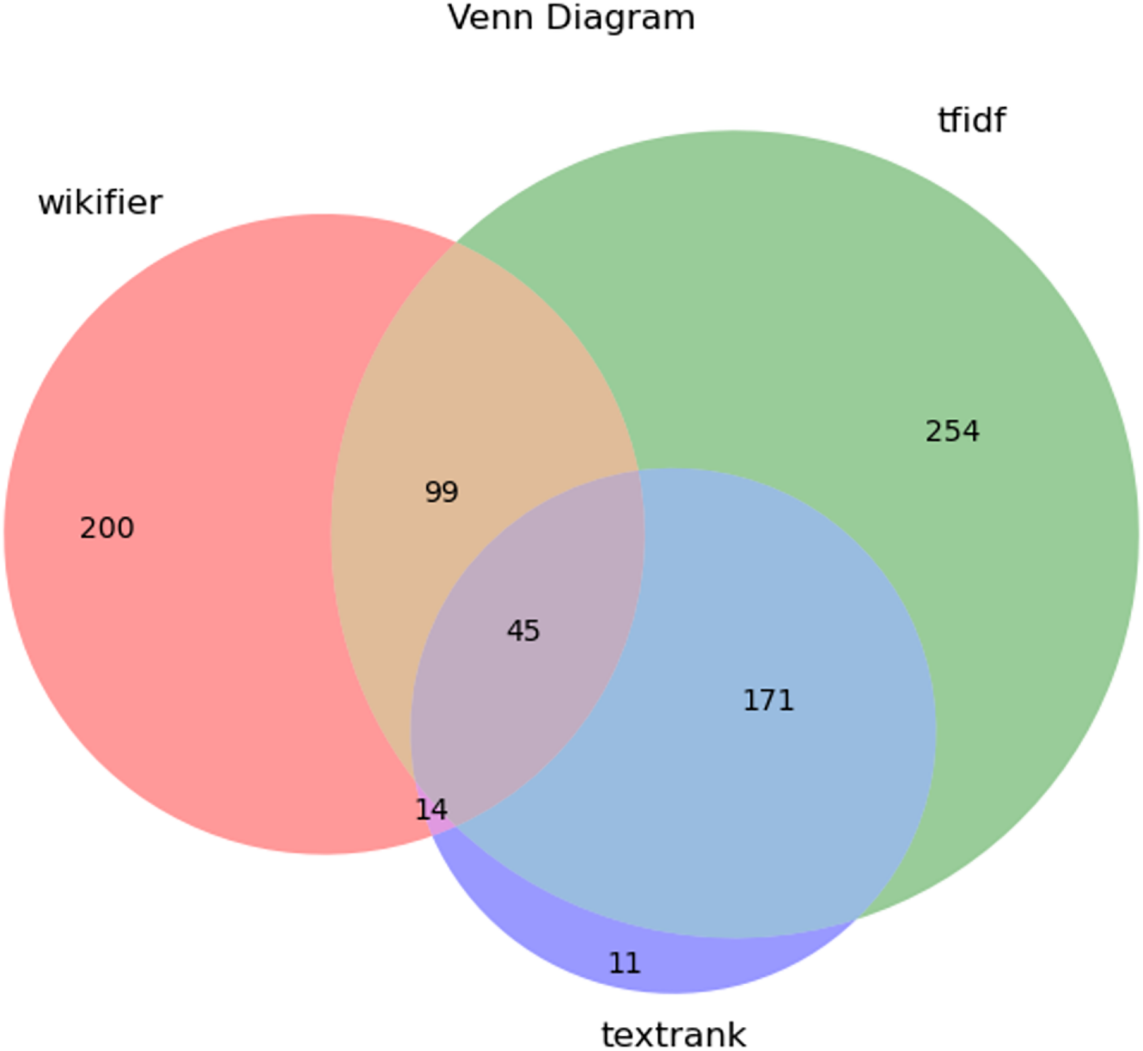
Venn diagram showing the overlap of key terms identified by TF-IDF, TextRank, and Wikifier.

The results presented in Fig. 7 allow us to assess the degree of agreement among the three methods to extract key terms. The highest number of unique terms was identified by the TF-IDF method (254 words), which can be explained by its statistical approach, where the importance of a word is determined by its frequency in the text relative to the entire *corpus*. Wikifier identified 200 unique terms, indicating its focus on conceptual connections with external knowledge bases rather than simple frequency analysis. TextRank, on the other hand, was the most conservative, extracting only 11 unique terms, as its algorithm is based on a graph model and identifies only the most significant words in terms of their interconnections.

The intersection of two methods also provides important insights. Specifically, 99 terms were simultaneously identified by both TF-IDF and Wikifier, indicating the presence of terms that are significant both in terms of statistical frequency and conceptual relevance. The methods TF-IDF and TextRank identified 171 common terms, highlighting their similarity in extracting key words despite their different approaches (statistics *vs*. graph-based model). The overlap between Wikifier and TextRank was minimal (14 terms), which can be explained by the fact that Wikifier relies on knowledge from external sources, while TextRank identifies key terms based on internal text relationships.

Of particular importance is the intersection of all three methods, where 45 terms overlapped. This number can be interpreted as an indicator of method agreement. Despite differences in algorithms, these words were recognized as important by all three approaches, confirming their special significance in the text. The higher this number, the more methods agree on the most important terms, indicating the high stability of these terms regardless of the extraction methodology. In our case, 45 is a relatively small proportion of the total number of words extracted, suggesting significant differences in the approaches of the methods. Thus, using multiple methods simultaneously provides a more comprehensive representation of key terms than applying a single method alone, as each contributes uniquely to text analysis.

#### Word clouds

**Figure 8:**
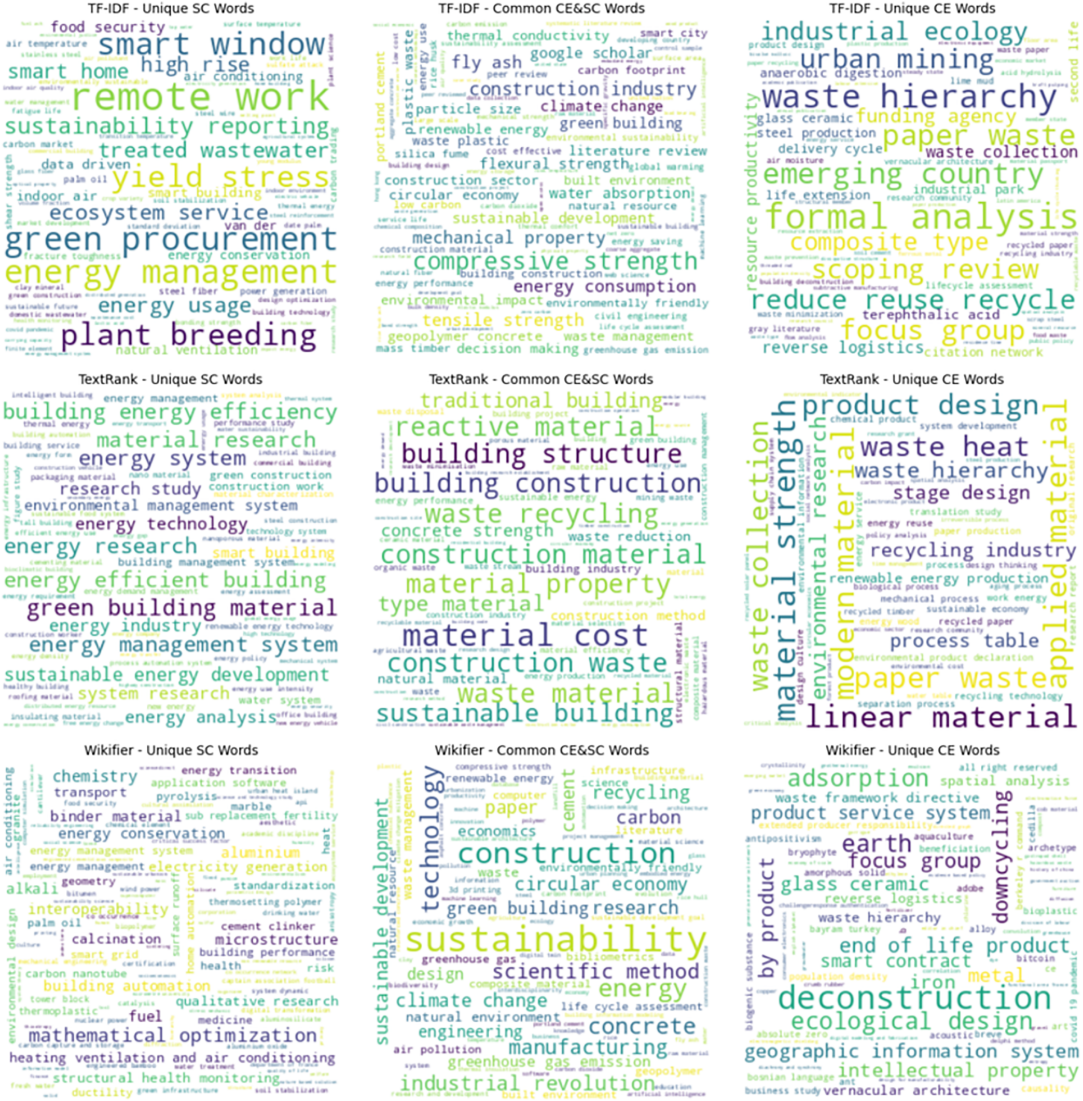
Word clouds visualising key terms extracted by TF-IDF, TextRank, and Wikifier, highlighting their overlaps and unique contributions.

To further compare the performance of TF-IDF, TextRank, and Wikifier in extracting key terms, word clouds were generated for each method (Fig. 8). A key observation is that TF-IDF tends to extract a broad set of terms, particularly those appearing frequently in the text. This method highlights both general and specific terms related to SC and CE. In particular, TF-IDF identifies a significant number of technical and engineering-related words, such as “*yield stress”*, “*composite strength”*, and “*mechanical properties”*. However, this statistical approach does not account for the contextual meaning of words, leading to the inclusion of some less conceptually relevant terms.

TextRank, in contrast, is more selective, extracting fewer but more contextually significant terms. The words identified by TextRank are often central to the thematic structure of the text, focusing on fundamental concepts such as “*building energy efficiency”*, “*construction material”*, and “*sustainable building”*. This reflects the method’s ability to capture interrelations between words through its graph-based ranking algorithm. However, TextRank appears to be more aligned with SC than CE, as fewer circular economy-specific terms emerge compared to TF-IDF and Wikifier.

Wikifier differs from both TF-IDF and TextRank by integrating external knowledge bases, resulting in a more conceptually rich set of extracted terms. This method emphasises broader sustainability concepts such as “*sustainability”*, “*carbon footprint”*, and “*industrial revolution”*, making it particularly effective in linking terms to well-established knowledge domains. Additionally, Wikifier identifies interdisciplinary terms such as “*intellectual property”* and “*geographical information system”*, which are not as prominent in the other methods. This suggests that Wikifier is better suited for capturing domain-specific knowledge beyond mere word frequency or internal text structure.

When comparing the agreement between methods, we observe that:

1. **TF-IDF and TextRank** share a notable number of key terms, particularly in the domain of construction materials and energy efficiency. This suggests that despite their methodological differences, both approaches recognise frequently occurring words that are also structurally important within the text.
2. **TF-IDF and Wikifier** exhibit strong overlap in statistical and conceptually relevant words, highlighting terms that are both frequent and knowledge-driven.
3. **TextRank and Wikifier**, however, have minimal intersection, as TextRank prioritises structural importance within the text, while Wikifier derives importance from external domain knowledge.

Overall, the word clouds demonstrate that each method brings own perspective to keyword extraction. While TF-IDF provides a broad statistical perspective, TextRank refines the selection by focusing on structurally significant words, and Wikifier extends the analysis by integrating external knowledge. The combination of these approaches allows for a more comprehensive understanding of key terms within the domains of SC and CC.

## Discussion

Based on the results of the three methodologies, it can be concluded that CC and SC are indeed similar terms with some overlapping topics (*e.g*., energy, materials, and lifecycle analysis), which aligns with the results of other studies (Sauvé, Bernard & Sloan, 2016; Hasheminasab et al., 2022). Nevertheless, there are certain differences as well. The focus of CC is more specific and practical, emphasising resource efficiency, waste management, and industry-specific processes. It targets the operational aspects of recycling and resource recovery within the construction industry. This focus on operational aspects of resource efficiency makes CC ideal for project-level implementation, directly reducing a construction project’s environmental footprint. In turn, SC’s scope is broader and more holistic, covering urban planning, community development, and long-term environmental impacts. It integrates various aspects of sustainability, including social and economic dimensions, into construction practices. These results find supports in the academic literature (Mahanty et al., 2021; Mirzyńska et al., 2021; Charef, Lu & Hall, 2022; Reimers & Gurevych, 2019; Hasheminasab et al., 2022; Ossio, Salinas & Hernández, 2023). SC develops sustainable practices throughout a project’s lifecycle by integrating social and economic considerations alongside environmental ones. This approach is valuable for large-scale planning and policy development within the construction industry.

TF-IDF provides a clear differentiation between the focused approach of CC and the comprehensive nature of SC. TextRank highlights the nested relationship between CE and sustainability, showing how CE principles contribute to broader sustainability goals. Concept Matrix demonstrates the contextual associations of key terms, revealing the operational focus of CE and the sector-specific approach to sustainability. Table 9 provides an overview of the comparison of their methods. The differences in results across the three methodologies stem from their distinct analytical approaches. TF-IDF identifies key terms based on their frequency within the dataset, effectively capturing commonly used terminologies but without considering their contextual relationships. In contrast, TextRank ranks terms based on their connectivity within the text, emphasising influential keywords that frequently co-occur, which can shift the focus toward interrelated concepts rather than purely frequent terms. Meanwhile, the Concept Matrix approach goes beyond term frequency and co-occurrence rankings by identifying associative patterns between concepts, explaining the strength of their relationships rather than their standalone prominence. As a result, each method highlights different aspects of CC and SC, enriching the analysis with complementary perspectives rather than a singular, uniform conclusion.

**Table 9:** Comparison of results from TF-IDF, TextRank, and semantic annotation approaches.

Methodology	Key themes in circular construction (CC)	Key themes in sustainable construction (SC)	Common themes	Notable differences
TF-IDF	Focus on resource efficiency, waste utilization, and recycling	Broad focus on urban planning, energy management, and sustainability	Environmental impact assessment, lifecycle analysis	CC emphasizes operational aspects; SC integrates social, economic, and environmental concerns
TextRank	Emphasises resource recovery and sustainability principles within CE	Highlights energy efficiency, building materials (*e.g*., timber), and regional focus	Lifecycle analysis, assessment methodologies	CC focuses on processes and industries; SC emphasises long-term urban sustainability
Semantic annotation	Reveals strong connections between sustainability and CE in built environment contexts	Sustainability shows higher conceptual integration across materials, governance, and planning	Built environment, urban ecology, and waste management appear in both	Sustainability is more holistically embedded in regulatory and systemic frameworks than CE

Previous works have demonstrated how NLP can uncover hidden patterns in large corpora related to sustainability and CE (Moghayedi et al., 2021). Grasping the differences between CC and SC is crucial for improving construction practices in the real world. When designing and planning, architects and engineers can better customise their methods by focusing on either CC or SC principles. For example, CC focuses on designing buildings that allow materials to be reused and easily taken apart, which often involves using prefabricated parts that can be recycled later. On the other hand, SC concentrates on energy efficiency and using renewable materials, which affects choices like how a building is oriented, the type of insulation used, and the energy systems installed.

However, our analysis reveals a gap in the intersection of these fields, particularly in construction. From a practical industry perspective, recognising these specific distinctions enables construction firms to develop strategies that are specifically aligned with circular or sustainable goals, potentially enhancing their project life-cycle management. For instance, circular approaches can be prioritised where direct material reuse or closed-loop processes are paramount, whereas sustainable approaches can guide broader system-wide planning and collaboration among various stakeholders, such as urban planners, facility managers, and municipal authorities, to achieve long-term socio-economic and environmental benefits. Clear definitions help tailor communication and educational efforts for everyone involved, including contractors, clients, and workers. When stakeholders understand whether a project follows circular or sustainable principles, they can set the right expectations, collaborate better, and stay aligned with the project’s main goals. Contractors and developers can translate the CC-specific clusters (*e.g*., “resource recovery,” “reverse logistics”) into material-passport requirements and deconstruction-ready design checklists, while SC clusters (*e.g*., “urban ecology,” “energy-efficient building”) inform whole-life-cycle cost modelling and ESG reporting.

In terms of policy implications, clearer differentiation between sustainability and CE can assist policymakers in crafting targeted regulations and standards. By tailoring policies to the specific focus of circularity (*e.g*., waste reduction measures and material passport requirements) *vs* sustainability (*e.g*., incentive frameworks for green infrastructure and social equity), regulatory bodies can more effectively align legal frameworks with measurable goals. Additionally, these insights can guide the allocation of public funding and the development of performance metrics that incentivise both circular construction and sustainable design in complementary ways. Regulators may map our term clusters against current building codes to spot gaps, such as missing incentives for urban agriculture or end-of-life material take-back, and draft targeted amendments.

For the academic community, this study’s unique contribution lies in demonstrating the power of NLP methods to discern and differentiate key concepts within a sector as complex as construction. Beyond the immediate focus on sustainability and CE, the replicable methodological framework outlined here could inform comparative analyses of other intersecting concepts in built-environment research (*e.g*., resilience, net-zero design, regenerative strategies). The NLP workflow itself can be re-run on new corpora (grey literature, regional languages) to track how policy shocks or technological breakthroughs shift the discourse, creating a longitudinal “early-warning” system for emerging priorities.

## Limitations and validity

The methodology applied in this study contains certain limitations. First, our dataset consists mainly of review articles published between 2021 and 2024. This temporal constraint could introduce bias and limit the historical scope of findings. The NLP techniques employed, including TF-IDF, TextRank, and semantic annotation, proved effective in identifying key terms and topics. However, both TF-IDF and TextRank are highly sensitive to text preprocessing. In particular, removing numerical data, while necessary for clarity in some respects, may have inadvertently discarded important contextual information that could affect the interpretation of term associations and relationships. Semantic annotation generation relies on an external knowledge base—Wikipedia, which is a common practice in information extraction techniques. While useful, this may limit analysis depth and domain-specificity. Currently, no domain-specific knowledge base comprehensively covers the latest trends in the construction industry. Finally, the models used for this analysis, while effective for handling large corpora, are based on predefined parameters that might not fully capture nuances specific to the construction sector. Further validation using more diverse datasets, alongside qualitative assessments, will be required to ensure robustness and broader applicability of our conclusions.

## Conclusion

This study systematically analysed the terms “sustainability” and “circular economy” (CE) within the construction sector using advanced NLP techniques (TF-IDF, TextRank, and semantic annotation). The results indicate that while both concepts share a common aim of reducing environmental impact, CE tends to emphasise project-level resource efficiency, focusing on closed material loops and waste reduction, whereas sustainability extends to broader systemic considerations such as urban planning, social equity, and governance. These distinctions highlight the need for policymakers to adopt targeted regulations that reflect the immediate material-focused initiatives of circular construction alongside the long-term societal goals inherent in sustainable development. Moreover, by clearly defining these terms and illustrating their overlaps and differences, this research provides a replicable framework that industry practitioners and academics can use to align project strategies with the appropriate conceptual model. Employing NLP in this comparative analysis not only streamlines large-scale literature reviews but also uncovers subtle terminological nuances that may remain hidden through traditional qualitative approaches. Consequently, stakeholders can better navigate the complexities of integrating both circular and sustainable principles into construction practices, thereby optimizing resource usage while bolstering long-term resilience. Notably, the unique research findings of this study lie in demonstrating how data-driven text analysis can systematically disentangle two closely related yet distinct frameworks in construction. While previous research has applied NLP techniques to sustainability (Olawumi & Chan, 2018) and circular economy (Mhlanga, Haupt & Loggia, 2024; Silva Martins et al., 2024) separately, no study has simultaneously examined their intersection within the construction industry.

This research contributes to the field by addressing this gap and demonstrating the potential of NLP for integrated sustainability and circularity analysis in the built environment. In particular, this work clarifies that circular construction addresses immediate resource loops and material efficiency, whereas sustainable construction integrates socio-economic factors and operates across multiple scales, from local projects to regional initiatives. In sum, applying a multi-method NLP pipeline uncovers how circular construction functions as an operational accelerator nested within the broader, multi-scalar agenda of sustainable construction.

While this study focuses on English-language review articles in construction engineering, future research could incorporate non-English publications, broader interdisciplinary sources, and a more diverse range of document types. Although the time period scope is anchored in 2021–2024 literature, the workflow is openly shared and easily extensible; thus, re-analysing earlier or subsequent corpora will test the durability of our clusters and expose temporal tipping points in construction discourse. Additionally, combining NLP with qualitative methods, such as expert interviews or stakeholder workshops, would enrich the findings by illuminating on-the-ground experiences, enabling more comprehensive guidance for advancing both sustainability and circularity in the construction industry.

## Appendix

### Reproducibility

**Dataset (DOI/URL):** https://data.mendeley.com/datasets/6w74d7x8s4/2 (DOI: 10.17632/6w74d7x8s4.2); DOI: 10.5281/zenodo.15510952, v1.0 AlexPak/paper-2024-nu-sustainable-constr.

**Computing infrastructure (operating system, hardware *etc*) requirements:**

All experiments were conducted on the following computing environment:

– CPU: 2 $\times$ Intel® Xeon® Gold 6354
– RAM: 128 GB DDR4
– GPU: 2 $\times$ NVIDIA A100 (40 GB each)
– Storage: High-speed NVMe SSD
– OS: Ubuntu 22.04 LTS
– Python: Version 3.10

**Software and dependencies:**

The analysis was implemented in Python using the following major packages:

$\bullet$ pandas $\bullet$ numpy $\bullet$ scikit-learn $\bullet$ nltk $\bullet$ matplotlib $\bullet$ networkx $\bullet$ gensim $\bullet$ transformers $\bullet$ sentence-transformers.

A full list of Python packages with pinned versions is provided in the accompanying requirements.txt file, which is submitted as supplementary material alongside this manuscript. This ensures full reproducibility of the results.

To install all dependencies, the following command can be used:

pip install -r requirements.txt

### Description of models used

**Justification for model type used:**

The selection of models and methods in this study is driven by the need to explore, analyze, and differentiate the nuances between circular construction and sustainable construction in the literature. Each model was carefully chosen to highlight different dimensions of this relationship:

- Concept Matrix approach: The Concept Matrix model was chosen to capture contextual associations between key terms, as it provides a detailed mapping of how circular construction is situated within the broader discourse on sustainability. This model allows for a comparison between the sector-specific focus of circular construction and the more general nature of sustainable construction.
- Clustering methods: Clustering was employed to group terms found in the literature and visually represent the conceptual landscape. This method was selected for its ability to reveal patterns and groupings in a way that is both interpretable and meaningful for understanding the relationship between different concepts in the dataset.
- TextRank algorithm: TextRank was selected due to its strength in ranking terms based on importance within a graph-based structure. This method highlights the hierarchical and nested relationships between circular economy and sustainability concepts, aligning with the study’s goal of showing how circular construction fits within broader sustainability frameworks.
- TF-IDF approach: The TF-IDF method was selected for its effectiveness in distinguishing between terms that are more specific to circular construction compared to those associated with sustainability. This aligns with the objective of pinpointing the more targeted nature of circular construction as compared to the broader scope of sustainable construction.
  Assessment metrics (Justification):
- NLP similarity: A measure of semantic similarity between terms, based on contextual embeddings. This helped identify closely related concepts within the *corpus*.
- Rank: For terms extracted *via* TextRank, a rank value was assigned based on their centrality in the term co-occurrence graph. Higher-ranked terms are considered more crucial for understanding the key topics in the text.
- Importance (TF-IDF): The TF-IDF scores were used to assess the relative importance of terms in individual documents. Terms with higher TF-IDF scores are indicative of their significance within a particular document or set of documents.
- Entropy: The entropy of the term distribution was calculated to assess the diversity of terms within the *corpus*. A higher entropy value indicates a wider variety of terms used across the dataset.
- Graph metrics: The TextRank graph was analyzed using various network-based metrics, including:
  - Node centrality: Identifies the most influential terms based on their connectedness in the co-occurrence network.
  - Degree: The number of direct connections a term has with other terms.
  - Clustering coefficient: Measures how tightly connected the neighbors of a term are, providing insights into local term clusters.

**Repository link:** https://github.com/AlexPak/paper-2024-nu-sustainable-constr.

This repository is organized into several Jupyter Notebooks that represent the different methods used in the study:

ConceptMatrix.ipynb: Implements the semantic annotation approach to discover contextual associations between key terms in the dataset, revealing how circular construction is more operational and sector-specific compared to sustainable construction.

Clustering.ipynb: Contains code for clustering the dataset based on the terms found using NLP methods, providing a clear visual representation of how different concepts are grouped within the literature.

Evaluation_Methods.ipynb: Includes the evaluation metrics used to assess the performance and relevance of the NLP methods in identifying key themes in the literature.

TextRank_approach.ipynb: Applies the TextRank algorithm to reveal the relationships between circular economy and sustainability concepts, showing the nested nature of circular economy principles within broader sustainability goals.

TFIDF_approach.ipynb: Demonstrates the use of the TF-IDF method to differentiate between the targeted approach of circular construction and the more comprehensive nature of sustainable construction.

## Supplemental Information

10.7717/peerj-cs.3085/supp-1 Supplemental Information 1 Versions of mentioned packages for full reproducibility. Versions of mentioned packages for full reproducibility.

10.7717/peerj-cs.3085/supp-2 Supplemental Information 2 Reproducibility.

10.7717/peerj-cs.3085/supp-3 Supplemental Information 3 Description of models used.

## List of abbreviations

AI: Artificial Intelligence
BERT: Bidirectional Encoder Representations from Transformers
CC: Circular Construction
CE: Circular Economy
GPT: Generative Pretrained Transformer
HTML: HyperText Markup Language
ISSAI: Institute of Smart Systems and Artificial Intelligence
NLP: Natural Language Processing
PDF: Portable Document Format
REIN: Rare Earth Innovation Network
SC: Sustainable Construction
TF: Term Frequency
TF-IDF: Term Frequency-Inverse Document Frequency
UOVN: Unified Open-Vocabulary Network
URL: Uniform Resource Locator
VOSviewer: Visualization of Similarities Viewer

## Additional Information and Declarations

### Data Availability

The following information was supplied regarding data availability:

The code and data are available at GitHub and Zenodo:

- https://github.com/AlexPak/paper-2024-nu-sustainable-constr.

- AlexPak. (2025). AlexPak/paper-2024-nu-sustainable-constr: Public Release (v1.0). Zenodo. https://doi.org/10.5281/zenodo.15510952.

The data for reproducibility is available at Mendeley: Karaca, Ferhat; Tleuken, Aidana; Varol, Huseyin Atakan; Akhmetov, Iskander; Pak, Aleksandr; Akzhalova, Assel; Aubakirov, Shakarim (2024), “Dataset | Sustainable and Circular Construction Terms ”, Mendeley Data, V2, doi: 10.17632/6w74d7x8s4.2.
