# Supplementary material for: Beyond buzzwords: NLP reveals common threads in sustainable and circular construction discourse
Source: PeerJ Comput Sci. 2025 Jul 31;11:e3085. doi: 10.7717/peerj-cs.3085 (PMC12453784; doi:10.7717/peerj-cs.3085)
Supplement: Supplemental Information 2 [file peerj-cs-11-3085-s002.pdf]

Reproducibility

Dataset (DOI/URL)

https://data.mendeley.com/datasets/6w74d7x8s4/2

Computing infrastructure (operating system, hardware etc)

Requirements

To run the notebooks in this repository, you will need the following Python packages:

- pandas
- numpy
- scikit-learn
- nltk
- matplotlib
- networkx
- gensim

To install the required dependencies, you can run:

pip install -r requirements.txt
