# Supplementary material for: Beyond buzzwords: NLP reveals common threads in sustainable and circular construction discourse
Source: PeerJ Comput Sci. 2025 Jul 31;11:e3085. doi: 10.7717/peerj-cs.3085 (PMC12453784; doi:10.7717/peerj-cs.3085)
Supplement: Supplemental Information 3 [file peerj-cs-11-3085-s003.pdf]

Description of models used:

Justification for Model Type Used:

The selection of models and methods in this study is driven by the need to explore, analyze, and differentiate the nuances between circular construction and sustainable construction in the literature. Each model was carefully chosen to highlight different dimensions of this relationship:

- **Concept Matrix Approach:** The Concept Matrix model was chosen to capture contextual associations between key terms, as it provides a detailed mapping of how circular construction is situated within the broader discourse on sustainability. This model allows for a comparison between the sector-specific focus of circular construction and the more general nature of sustainable construction.
- **Clustering Methods:** Clustering was employed to group terms found in the literature and visually represent the conceptual landscape. This method was selected for its ability to reveal patterns and groupings in a way that is both interpretable and meaningful for understanding the relationship between different concepts in the dataset.
- **TextRank Algorithm:** TextRank was selected due to its strength in ranking terms based on importance within a graph-based structure. This method highlights the hierarchical and nested relationships between circular economy and sustainability concepts, aligning with the study's goal of showing how circular construction fits within broader sustainability frameworks.
- **TF-IDF Approach:** The TF-IDF method was selected for its effectiveness in distinguishing between terms that are more specific to circular construction compared to those associated with sustainability. This aligns with the objective of pinpointing the more targeted nature of circular construction as compared to the broader scope of sustainable construction.

Assessment Metrics (Justification):

- **NLP Similarity**: A measure of semantic similarity between terms, based on contextual embeddings. This helped identify closely related concepts within the corpus.
- **Rank**: For terms extracted via TextRank, a rank value was assigned based on their centrality in the term co-occurrence graph. Higher-ranked terms are considered more crucial for understanding the key topics in the text.
- **Importance (TF-IDF)**: The TF-IDF scores were used to assess the relative importance of terms in individual documents. Terms with higher TF-IDF scores are indicative of their significance within a particular document or set of documents.
- **Entropy**: The entropy of the term distribution was calculated to assess the diversity of terms within the corpus. A higher entropy value indicates a wider variety of terms used across the dataset.
- **Graph Metrics**: The TextRank graph was analyzed using various network-based metrics, including:
  - **Node Centrality**: Identifies the most influential terms based on their connectedness in the co-occurrence network.
  - **Degree**: The number of direct connections a term has with other terms.

    - **Clustering Coefficient**: Measures how tightly connected the neighbors of a term are, providing insights into local term clusters.

The Concept Matrix evaluation relies on three main metrics.

- First, co-occurrence of concepts measures how often specific terms appear together within the text. This helps to identify relationships between key concepts such as "sustainability" and "circular economy." The co-occurrence values are normalized to allow for comparison between different terms.
- Second, Spearman and Pearson correlations are used to assess the strength of relationships between concept pairs. Pearson correlation evaluates linear relationships, while Spearman correlation focuses on the rank of concept occurrences. Both provide insights into how closely related certain concepts are in the literature.
- Finally, keyword interactions examine how key concepts are associated with other terms. For instance, terms like "sustainability" are often linked with "construction" and "technology," while "circular economy" is associated with "recycling" and "waste management." These metrics offer a clear understanding of how concepts are connected and their thematic relevance in the analyzed texts.
